# Validity and Responsiveness of the Generic Health-Related Quality of Life Instrument (VetMetrica™) in Cats With Osteoarthritis. Comparison of Vet and Owner Impressions of Quality of Life Impact

**DOI:** 10.3389/fvets.2021.733812

**Published:** 2021-09-30

**Authors:** E. Marian Scott, Vinny Davies, Andrea M. Nolan, Cory E. Noble, Nathalie J. Dowgray, Alexander J. German, M. Lesley Wiseman-Orr, Jacqueline Reid

**Affiliations:** ^1^School of Mathematics and Statistics, University of Glasgow, Glasgow, United Kingdom; ^2^School of Computing Science, University of Glasgow, Glasgow, United Kingdom; ^3^Edinburgh Napier University, Edinburgh, United Kingdom; ^4^NewMetrica Ltd., Glasgow, United Kingdom; ^5^International Cat Care, Tisbury, United Kingdom; ^6^Faculty of Health and Life Sciences, University of Liverpool, Neston, United Kingdom; ^7^School of Education, University of Glasgow, Glasgow, United Kingdom; ^8^School of Veterinary Medicine, University of Glasgow, Glasgow, United Kingdom

**Keywords:** health–related quality of life, construct validity, responsiveness, osteoarthritis, owner opinion, known groups, convergent validity, co-morbidities

## Abstract

Validity is not an inherent property of a measurement scale and so evidence for validity relating to its use for particular purposes, with defined populations and in specified contexts must be accumulated. We have published the development of a web-based, generic health-related quality of life instrument (VetMetrica™) to measure the affective impact of chronic disease in cats and provided evidence for its validity in a mixed population of cats, some of which, according to veterinary judgement, were healthy and others of which were suffering from chronic conditions likely to affect their quality of life, often with multiple co-morbidities present. The first aim of the current study was to demonstrate the construct validity of the VetMetrica™ generic instrument when used with cats suffering from osteoarthritis, by testing the hypothesis that the health-related quality of life profile of cats with different severities of osteoarthritis would differ and by demonstrating convergent validity between the health-related quality of life profile scores and independently quantified vet-assessed pain and quality of life impact scores. The latter involved simple correlation analysis and investigation of the relationship between health-related quality of life domain scores and vet-assessed scores, when adjusted for other potential explanatory variables including number of comorbidities and age. Responsiveness—the ability to detect clinically relevant change—is an essential quality for an evaluative instrument and it also provides evidence for “longitudinal validity”. Therefore, a second aim of this study was to demonstrate that changes in health-related quality of life domain scores concurred with the clinician's impression of change over time in the health status of cats with osteoarthritis, thus providing evidence for the instrument's responsiveness. Previously, we have reported disagreement between owner and vet impression as to health status in cats in general, but not in relation to any specific disease. Accordingly, the third study aim was to investigate the extent of agreement or disagreement between owner impression of the impact of osteoarthritis on their cats' quality of life and vet impression of such impact. Fifty one percentage of cat owners believed their cats to be perfectly healthy despite a clinician diagnosis of osteoarthritis

## Introduction

Osteoarthritis (OA) is a common painful chronic disease in cats with a greater prevalence than was previously recognised (1). Its effective management is underpinned by the ability to measure pain reliably, and thus suitable measures of clinical impact are required. Such instruments should monitor pain effectively in an individual, enabling the selection of treatment with known efficacy. Several owner-report clinical metrology instruments have been developed (2, 3), which measure the functional limitations caused by OA, whilst there has also been interest in developing activity monitors for cats (4). However, the contemporary approach to pain measurement focuses on its affective dimension, which describes pain's unpleasantness i.e., the unpleasant feelings that are experienced which cause the suffering associated with pain (5). It has been suggested that a more comprehensive understanding of the affective component may be of fundamental importance to the development of treatments for chronic and neuropathic pain (5). Given that chronic pain in people interacts in a complex way with a person's emotional (social and psychological) and physical well-being, many human chronic pain instruments primarily measure the impact of the pain on quality of life (QOL) (6–8). Similarly, for veterinary orthopaedic studies, guidelines have been published that recognise the important contribution of the latter, given the recommendation that at least one owner-reported QOL instrument should be included, alongside at least one validated functional outcome (such as activity monitors and clinical metrology instruments) (9).

Quality of life is, like pain, a multi-dimensional construct that is subjectively experienced. We have previously defined animal QOL as an individual's evaluation of its circumstances, which results in or includes an affective state (10) and health-related quality of life (HRQL) as the subjective evaluation of circumstances that include altered health state and related interventions (11). Instruments designed to measure HRQL can be disease-specific, designed for particular conditions and their treatment, or they can be generic, designed for use in a variety of contexts in which health is good or is poor for a variety of reasons, see (12) for a comprehensive list. Such instruments either generate a single index score, which indicates that a patient is better or worse (12, 13) or a profile of scores which offers more information and may be more sensitive to group differences and to changes in health status over time (12, 14).

Previously, we have published the development, initial validation and reliability of a web-based, generic HRQL instrument (VetMetrica™) to measure the affective impact of chronic disease in cats (15). This structured questionnaire instrument is presented in an online survey format and takes the owner, on average, 5 min to complete. It contains 20 behaviour-based items to which the cat owner responds using a 7-point Likert scale (0 = could not be less to 6 = could not be more), enabling a profile of scores in 3 HRQL domains of Vitality, Comfort and Emotional wellbeing (EWB) to be generated. Subsequently, we have modified the scoring in three ways to improve interpretability: by normalising the scores to be centred at the healthy population average, by determining a score cut-off point that allows the user to classify a cat as sick or healthy, and by calculating the Minimal Important Difference (MID) in the normalised score to define a clinically significant improvement in each domain (16).

Validity is the most important property of a measurement instrument. It provides evidence that the instrument is able to measure the construct that it was intended to measure. Instrument developers should seek evidence for validity of three principal kinds: content, criterion and construct validity (17, 18). Criterion validity is demonstrated by the agreement of a new instrument with some existing “gold standard”, but a suitable gold standard does not always exist as was the case here. In our earlier study (15) we reported evidence for the content validity of the VetMetrica™ instrument which was determined using content validity indices for relevance (CVI_R)_ and clarity (I-CVI_C_) of each item, and some initial evidence for the instrument's construct validity by testing the instrument using a cohort of healthy cats and cats whose medical condition was deemed by the attending clinician to affect their QOL. Construct validation in that study (15) used factor analysis (FA) and hypothesis testing using “known groups” (17, 19, 20) (the hypotheses being that the HRQL profile of scores would differ between healthy cats and sick cats, and that the HRQL profile would be worse for cats with poorer health status defined by the number of co-morbidities present in individuals). In addition to such FA and “known groups” approaches to construct validation, developers can also seek evidence for convergent validity, a subtype of construct validity, where two measures that theoretically should be related to each other are shown to be so related (21).

Validity is the most important quality of any measurement instrument, and a body of evidence for validity must be accumulated in relation to its use for particular purposes, with defined populations and in specified contexts (22). Some evidence for the validity of the VetMetrica™ instrument has already been provided for the measurement of HRQL in a mixed population of cats, some of which, according to veterinary judgement, were healthy and others of which were suffering from chronic conditions likely to affect their QOL, often with multiple co-morbidities present. Generic instruments have been shown to perform satisfactorily for specific disease states in people, such as Crohn's Disease (23), OA (24), cardiac disease (25) and asthma (26), and generic instruments to measure QOL in animals should be able to be used in a similar way, with appropriate validation.

Consequently, the first objective of the current study was to seek evidence for the construct validity of the VetMetrica™ generic instrument when used with cats suffering from OA, by testing the hypothesis that the HRQL profile of cats with different severities of OA would differ and by demonstrating convergent validity between the HRQL profile scores and independently quantified vet-assessed pain and QOL impact scores. Convergent validity was demonstrated both by simple correlation analysis and by investigating the relationship between HRQL domain scores and vet-assessed scores, when adjusted for other potential explanatory variables including number of comorbidities and age.

Whilst the most important property of a scientifically robust health measurement instrument is validity, responsiveness—the ability to detect clinically relevant change—is an essential practical quality for an evaluative instrument and has been suggested to also provide evidence for “longitudinal validity” (27). Therefore, a second objective of this study was to demonstrate that changes in HRQL domain scores concurred with the clinician's impression of change over time in the health status of cats with OA, thus providing evidence for the instrument's responsiveness.

Previously, we have reported disagreement between owner and vet impression as to health status in dogs (28) and cats in general (15), but not in relation to any specific disease. In the latter publication the results of two independent field tests showed that 29 and 26% of owners of cats, deemed to be unhealthy following clinical assessment, believed their cats to be in perfect health. Accordingly, the third study objective was to investigate the extent of agreement or disagreement between owner impression of the impact of OA on their cats' QOL and vet impression of such impact.

## Methods

### Data Collection for Objectives 1, 2, and 3

The University of Glasgow School of Veterinary Medicine Ethics and Welfare Committee approved the work and written informed consent was obtained from the owners for the participation of their animals in these studies.

Data were collected from owners of 56 cats with a diagnosis of OA attending first opinion practises, feline specialist practises, and the University of Glasgow Small Animal Hospital (UGSAH) for previously conducted field studies relating to the feline VetMetrica™ HRQL instrument (15). Additionally, data were collected from 84 cats diagnosed with OA attending the Royal Canin Healthy Ageing Clinic, University of Liverpool and participating in the Cat Prospective Ageing and Welfare Study (CatPAWS). The latter is a longitudinal cohort study, established in 2016, to monitor pet cats during the ageing process. Cats are enrolled from the ages of 7–10 years and then followed prospectively by health evaluations on a biannual basis. Data are collected on physical, biochemical and clinical parameters, as well as QOL. All 140 owners completed at least one VetMetrica™ HRQL assessment online. In addition to the 20 questions comprising the VetMetrica™ instrument, owners were asked the following questions, personalised to their cat, in order to ascertain the owners' impression of their cat's QOL:

1. How would you rate (your cat's) quality of life?—very poor/poor/good/very good.
2. Is (your cat) currently in perfect health?—Yes/No.
3. If not, how much do you think (your cat's) health status is affecting the quality of life?—not at all/a little/somewhat/a lot.

These questions were independent of the 20 items comprising the HRQL assessment.

Concurrently, the attending vet was asked to complete a veterinary assessment comprising a list of common feline diseases, from which they recorded the presence and severity (mild/moderate/severe/end stage) for each cat. A freeform box allowed them to add a disease not specified in the list. They were also asked to complete the following questions:

1. On a scale of 0–10 with 0 being no impact and 10 being the most impact, please assess how much the cat's health status is reducing its quality of life (QoL):
No impact 0 1 2 3 4 5 6 7 8 9 10 Impact could not be greater.

2. On a scale of 0 to 10 with 0 being no pain and 10 being the pain could not be worse, please indicate what amount of pain you feel the cat is suffering?
No pain 0 1 2 3 4 5 6 7 8 9 10 Pain could not be worse.

3. How has the cat's general health status changed since the previous consultation?
Much worse/worse/unchanged/better/much better.
All data are available in the Supplementary Material.

### Objective 1–Construct Validity

Evidence for construct validity was sought using “known groups” and convergent validity approaches.

#### Statistical Analysis

The first owner and vet assessment pair were used for the statistical analysis. Data were analysed using Minitab^®^ 19 Statistical Software (2010) (www.minitab.com) and with an open-source statistical software environment (R, version 4.0.2, R Foundation for Statistical Computing, Vienna, Austria).

The level of statistical significance was set at 5% (*p* < 0.05) for all analyses.

#### Known Groups Validity

A one-way ANOVA was used to determine whether the difference between known groups (mild OA and moderate/severe OA) was statistically significant. Our prior hypothesis was that HRQL domain scores would vary between groups such that those for cats with mild OA would be greater than those for cats with moderate/severe OA HRQL scores, since higher scores represent better HRQL.

#### Convergent Validity

A Pearson correlation coefficient was used to determine the correlation (and convergent validity) of the 3 HRQL domain scores (Vitality, Comfort and EWB) and the vet-assessed QOL impact and pain scores. Further, linear models were fitted to explore the effects of age and other co-morbidities on the relationship between the vet-assessed QOL impact and pain scores, and the HRQL domain scores. In each case, the response was the vet-assessed score (QOL impact or pain), and the explanatory variables were the HRQL domain scores, age and number of co-morbidities. Non-significant explanatory variables (*p* > 0.05) were removed and the final model was reported.

### Objective 2: Responsiveness

#### Statistical Analysis

Using the 140-cat data set for Objective 1 where the owner had completed multiple assessments and where an available paired veterinary assessment was completed within ≤14 days of each owner assessment, change in HRQL scores was calculated as the difference between two consecutive observations. To assess whether the change in HRQL score reflected the direction of change in vet-assessed pain and QOL impact, individual *t*-tests were carried out for each HRQL domain within each direction of change (decrease, no change and increase) for vet-assessed pain and QOL impact scores. In the case of a decrease in the vet-assessed scores, a one-sided *t*-test was used with the hypothesis that there would be a positive change in HRQL domain score. Similarly, a one-sided *t*-test was used in the case of an increase in the vet-assessed scores with the hypothesis that there would be a negative change in HRQL domain score. Finally, a two-sided *t*-test was used in the case of no-change in the vet-assessed scores. In each case the significance level was set at 5%, with a *p* < 0.05 indicating a significant relationship. For one-sided analyses, the confidence intervals may be unbounded, and the results in this case are presented as either >*limit* or <*limit*.

### Objective 3–Agreement Between Veterinarian and Owner Global Assessments of QOL

Boxplots were constructed to examine the relationship between the veterinarian's assessment of impact on QOL and the owner's impression of their cat's QOL, and between the veterinarian's assessment of impact on QOL and the owner's impression of how much their cat's health status was impacting its QOL. Pairwise comparisons between groups were assessed using two sample *t*-tests (1 and 2 -sided) and 95% confidence intervals.

## Results

### Objective 1

**Figure 1:**
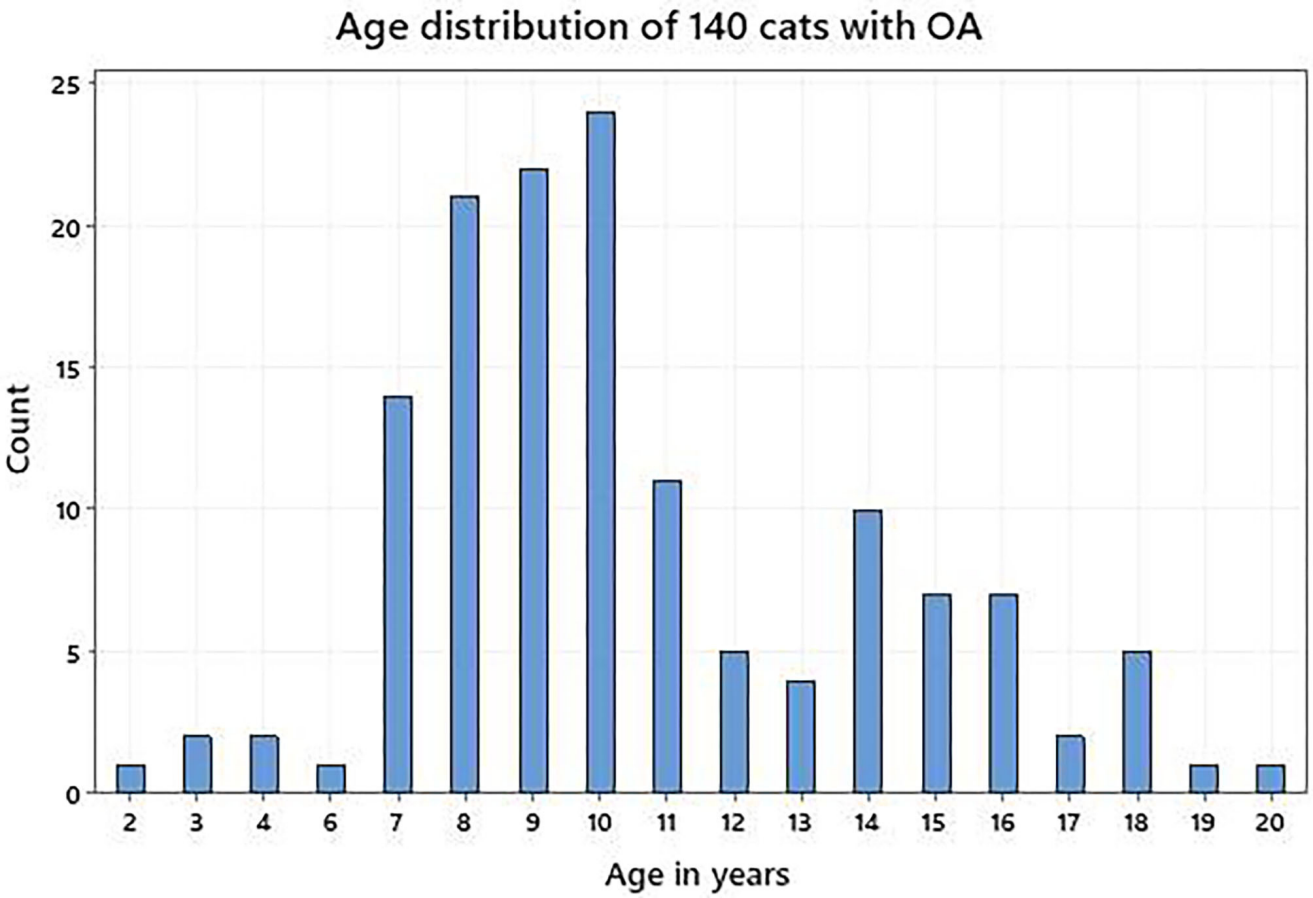
Age distribution of 140 cat subjects with osteoarthritis.

Owners of 140 cats, median age 10 years (range, 2–20 years; Figure 1) completed a first assessment; 80 were female, 60 were male and most were neutered, with various breeds represented (Table 1).

**Table 1:** Distribution of breeds for study cats (*N* = 140).

**Breed**	**Number of cats**
Domestic Short Hair	100
Domestic Medium Hair	3
Domestic Long Hair	10
Siamese	10
Burmese	2
British Short Hair	5
Bengal, Ragdoll, Persian Long Hair, Persian, Oriental Short Hair, Ocicat, Maine Coon, European Short Hair, Egyptian Mau, Balinese	One of each breed

#### Known Groups Validity

**Figure 2:**
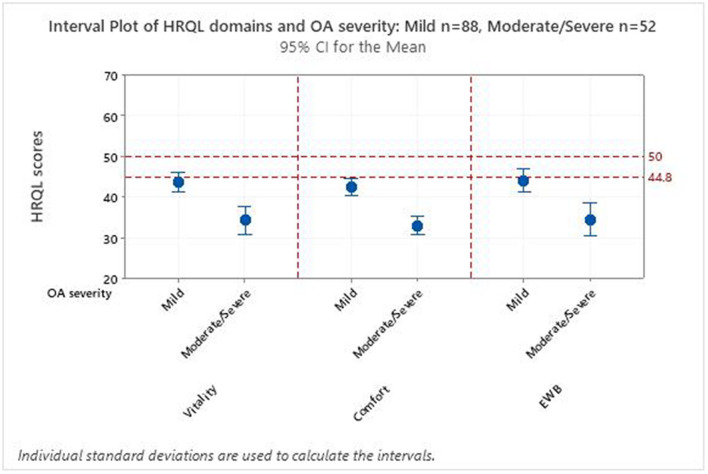
Interval plot of the mean and confidence intervals for health-related quality of life (HRQL) scores in Vitality, Comfort and Emotional Wellbeing (EWB) domains for 140 cats with mild (*n* = 88) or moderate/severe (*n* = 52) osteoarthritis. The dotted line indicates the score (50) which represents the healthy population average while the broken line represents the threshold score (44.8) above which 70% of healthy cats lie.

There were only nine occasions when cats were considered to have severe OA so these were included with the moderately affected cats to form a moderate/severe group. Figure 2 is an interval plot of the mean and confidence intervals for HRQL scores in Vitality, Comfort and EWB for 140 cats with mild or with moderate/severe OA as determined by an attending veterinarian. All the mean scores were <50.0, representing the healthy population average, whilst all mean scores were <44.8 above which 70% of healthy cats will score (14). For all three domains, the mean HRQL domain score was less for cats in the moderate-severe OA group compared with the mild OA group (Table 2; *p* < 0.001 for all), providing evidence for construct validity.

**Table 2:** Descriptive statistics for three HRQL domains, vitality, comfort and emotional wellbeing (EWB) for mild (*n* = 88) and moderate/severe (*n* = 52) groups of cats affected by OA.

**Domain**	**Severity**	**Mean**	**SD**	**Minimum**	**Q1**	**Median**	**Q3**	**Maximum**
Vitality	Mild	43.57	11.60	11.47	37.92	44.16	52.15	63.99
	Moderate/severe	34.21	12.11	0.05	28.24	34.42	43.56	56.07
Comfort	Mild	42.45	10.25	22.02	34.74	40.15	51.40	59.55
	Moderate/severe	32.91	8.09	15.71	27.53	31.47	37.00	59.55
EWB	Mild	44.09	13.34	8.26	34.59	47.34	55.02	58.84
	Moderate/severe	34.41	14.67	0.00	28.67	36.32	45.97	58.84

#### Convergent Validity

Seventeen veterinarians did not complete QOL impact and pain scores. Consequently this analysis was conducted on results from 123 cats. There were weak negative associations (29) between both the veterinary pain and QOL score and all three HRQL domains (Table 3; *p* < 0.001 for all). These statistically significant negative correlations indicated that as vet-assessed pain and QOL impact scores increased, the HRQL domain scores decreased, providing additional evidence for construct validity (Table 3).

**Table 3:** Pearson correlation coefficients and respective *P* values for the reported comparisons between Vitality, Comfort and Emotional Wellbeing (EWB), and the vet-assessed pain and QOL impact scores.

	**Vet QOL impact score**	**Vet pain score**
Vitality	−0.35 (*p* = <0.001)	−0.35 (*p* = <0.001)
Comfort	−0.33(*p* = <0.001))	−0.39 (*p* = <0.001)
EWB	−0.22 (*p* = 0.017)	−0.26 (*p* = 0.004)

#### Effects of Age and Other Co-morbidities on the Relationship Between the Vet-Assessed QOL Impact and Pain Scores and the HRQL Domain Scores

Apart from 11 cats that had OA with no co-morbidities (CM) reported, the remainder of the cats suffered from a variety of co-morbidities (median 2, range 0–7), the most commonly reported being dental disease, chronic kidney disease and other chronic medical conditions, which the veterinarians were not asked to specify. For the purpose of the analysis the comorbidity score was categorised as either ≤3 and >3 comorbidities.

A separate regression model for vet-assessed QOL impact and pain scores with each HRQL domain score, age and number of comorbidities was first fitted, and then variable selection (removal of all explanatory variables which were not statistically significant) carried out. For vet-assessed QOL impact, the categorised number of co-morbidities and each HRQL domain score were found to be statistically significant (Table 4). The mean vet-assessed QOL impact score was higher where there were more than three co-morbidities, and as each HRQL score increased, the QOL impact score decreased. Overall, between 10 and 14% of the variation in QOL impact was explained. For vet-assessed pain scores, age was statistically significant, as was the categorised number of co-morbidities. As the HRQL domain score increased when accounting for age, the pain score decreased, as age increased so did the pain score, and pain scores were on average higher in the >3 co-morbidities category. Overall, between 17 and 20% of the variation in pain score was explained by age, co-morbidities and the HRQL domain score.

**Table 4:** Results from regression model fitted with vet-assessed QOL impact and pain scores, and HRQL domains Vitality, Comfort and Emotional wellbeing (EWB), taking into account age (in years) and the number of co-morbidities (CM) present.

**Response**	**Vitality (*p*-value)**	**Comfort (*p*-value)**	**EWB (*p*-value)**
Vet pain score	Vitality: −0.039 (0.017)	Comfort: −0.059 (0.003)	EWB: −0.028 (0.04)
	Age: 0.123 (0.03)	Age: 0.102 (0.07)	Age: 0.135 (0.017)
	CM: 0.899 (0.06)	CM: 0.936 (0.045)	CM: 1.145 (0.015)
Vet QOL impact score	Vitality: −0.054 (0.003)	Comfort: −0.062 (0.005)	EWB: −0.031 (0.048)
	CM: 1.23 (0.023)	CM: 1.34 (0.011)	CM: 1.652 (0.002)

These regression analyses reveal anticipated relationships between HRQL domain scores and vet-assessed QOL impact and pain, after accounting for age and number of co-morbidities, which provides further evidence of convergent validity and so for the construct validity of the instrument.

### Objective 2

In seven cats date-matched observations were not available: for the remaining 133 cats there were 267 matched (owner and vet) observations. There were a total of 134 change measurements (from 74 cats) where two consecutive matched observations met the 14-day interval criterion. Thirty-seven of these cats had one change measurement, 21 had two, nine had three and seven had four. The average time between vet visits for cats with more than one change measurement was 176 days.

**Figure 3:**
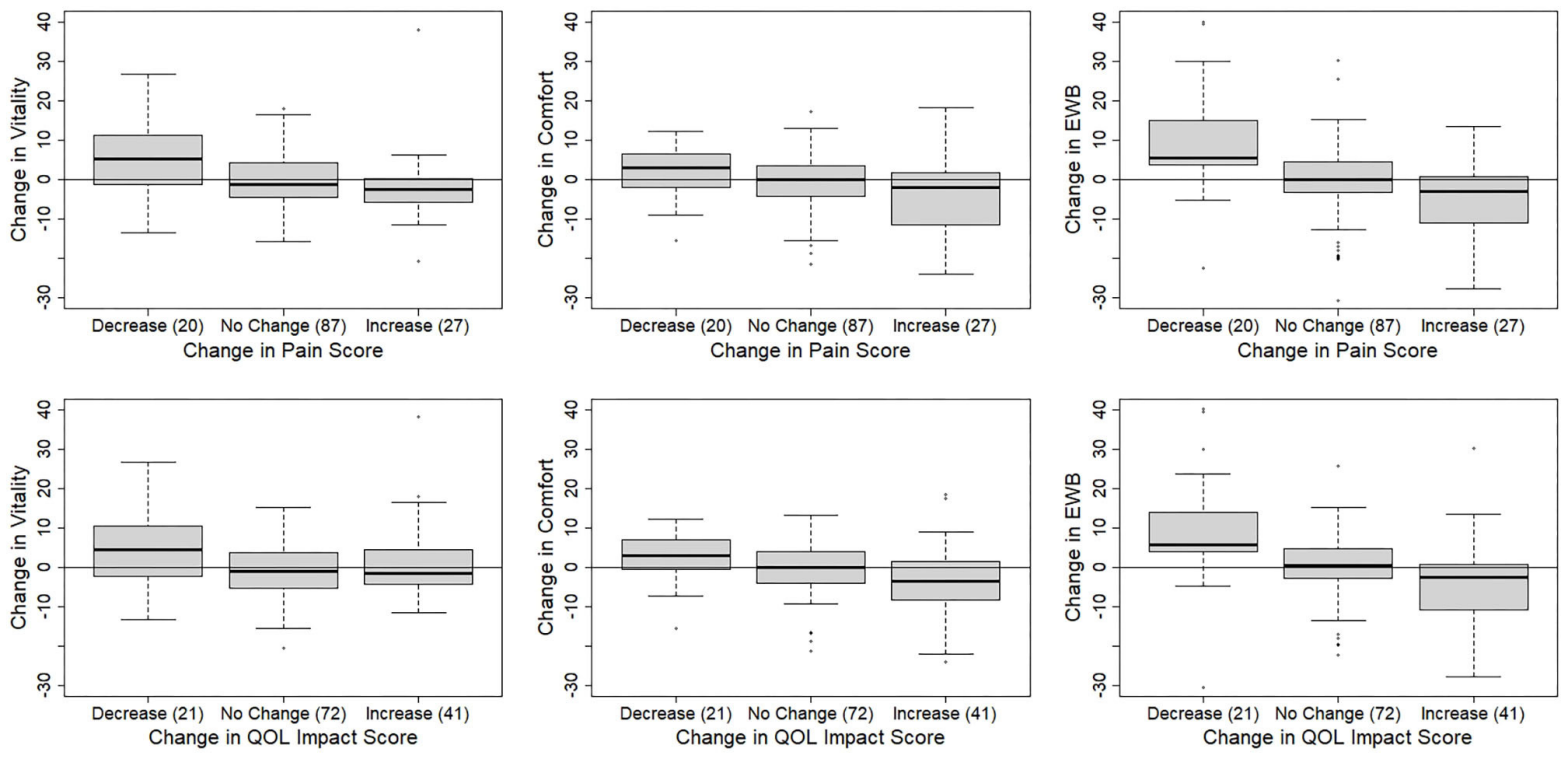
Boxplots of change in the three health related quality of life (HRQL) domain scores according to change in 134 vet-assessed quality of life (QOL) impact and pain scores from 74 cats.

The results for the change in HRQL domain scores for each type of change in vet-assessed pain and QOL impact are shown in Figure 3; Table 5. For vet-assessed pain score, there were no significant changes in any HRQL domains in cats where no change in pain score was reported on the vet assessment. However, in cats where pain score increased, HRQL domain scores decreased for Comfort (*p* = 0.015) and EWB (*p* = 0.022) but not Vitality (*p* = 0.194), whereas, in cats where pain score decreased, HRQL scores for Vitality (*p* = 0.009) and EWB increased (*p* = 0.004), but there was no change in Comfort score (*p* = 0.145). For the vet-assessed QOL impact score, there also were no significant changes in any HRQL domains in cats where no change in veterinary score was reported. Further, in cats whose vet assessment QOL impact score increased, domain scores for Comfort (*p* = 0.011) and EWB (*p* = 0.016), but not vitality (*p* = 0.736), decreased; conversely, for cats whose vet-assessed QOL impact score decreased, domain scores for Vitality (*p* = 0.018) and EWB (*p* = 0.004) increased but the domain score for comfort did not (*p* = 0.137).

**Table 5:** Changes in health-related quality of life domain scores, Vitality, Comfort and Emotional Wellbeing (EWB) with respect to change in vet-assessed pain score (VAP) and vet-assessed QOL impact score (VQOLI).

**HRQL Score**	**Decrease in VAP**		**No Change in VAP**		**Increase in VAP**	
	***p***	**Mean (95% CI)**	***p***	**Mean (95% CI)**	***p***	**Mean (95% CI)**
Vitality	0.009	6.35 (>2.15)	0.891	0.10 (−1.33, 1.53)	0.194	−1.64 (<1.55)
Comfort	0.145	1.68 (>-0.99)	0.379	−0.65 (−2.12, 0.81)	0.015	−4.43 (<-1.14)
EWB	0.004	9.90 (>4.23)	0.597	−0.55 (−2.63, 1.52)	0.022	−3.88 (<-0.75)
**HRQL Score**	**Decrease in VQOLI**		**No Change in VQOLI**		**Increase in VQOLI**	
	***p***	**Mean (95% CI)**	***p***	**Mean (95% CI)**	***p***	**Mean (95% CI)**
Vitality	0.018	5.35 (>1.27)	0.317	−0.80 (−2.39, 0.78)	0.736	0.89 (<3.24)
Comfort	0.137	1.78 (>-0.95)	0.471	−0.58 (−2.19, 1.02)	0.011	−3.37 (<-0.99)
EWB	0.004	9.96 (>4.23)	0.891	−0.14 (−2.12, 1.85)	0.016	−3.76 (<-0.92)

*Values represent mean (and 95% confidence intervals) change in HRQL domain score, along with the associated P values*.

### Objective 3

**Figure 4:**
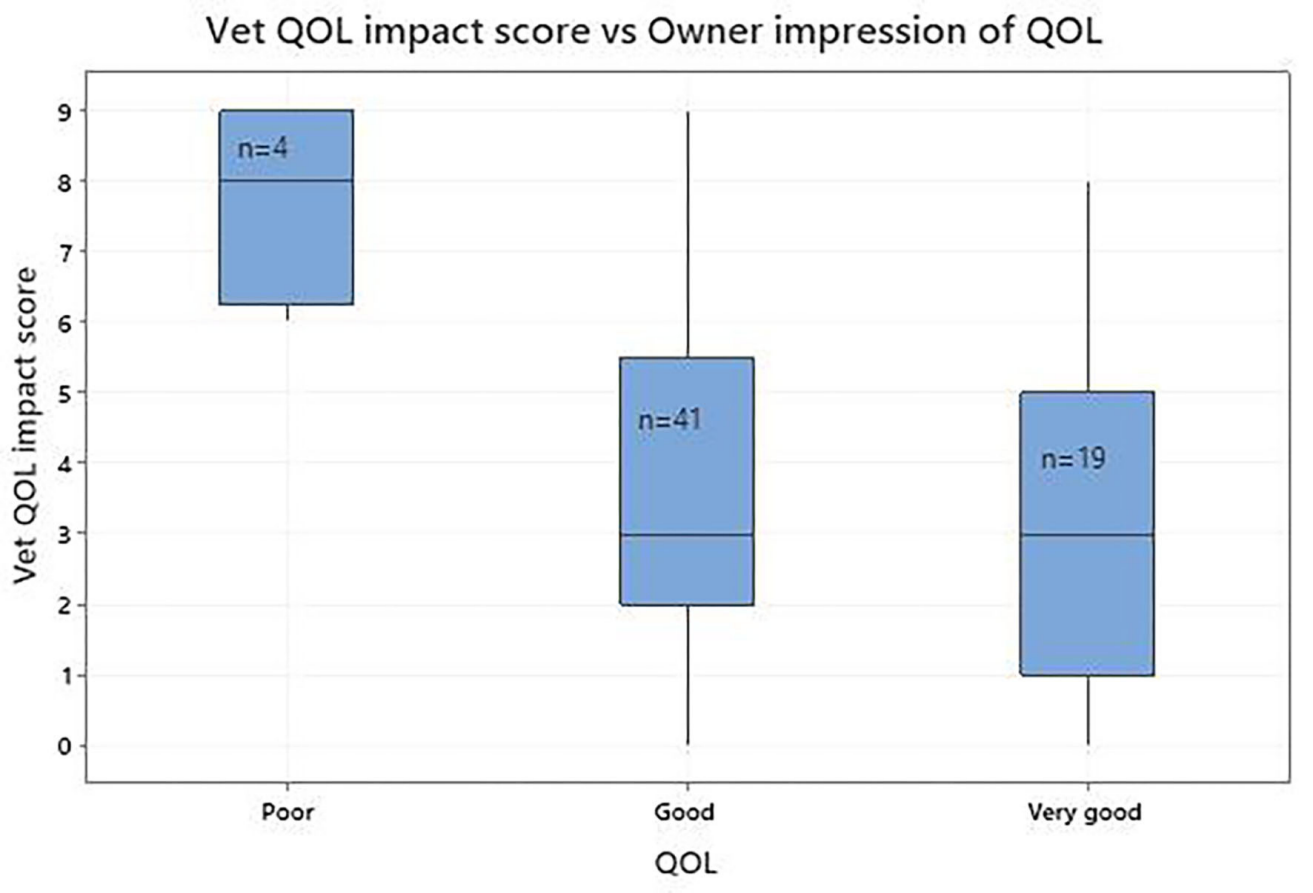
Boxplot of the vet-assessed quality of life (QOL) impact score (scale 0–10, where 0 is no impact and 10 is maximum possible impact) and the owner's impression of their cat's QOL (poor/very poor; good; very good).

**Figure 5:**
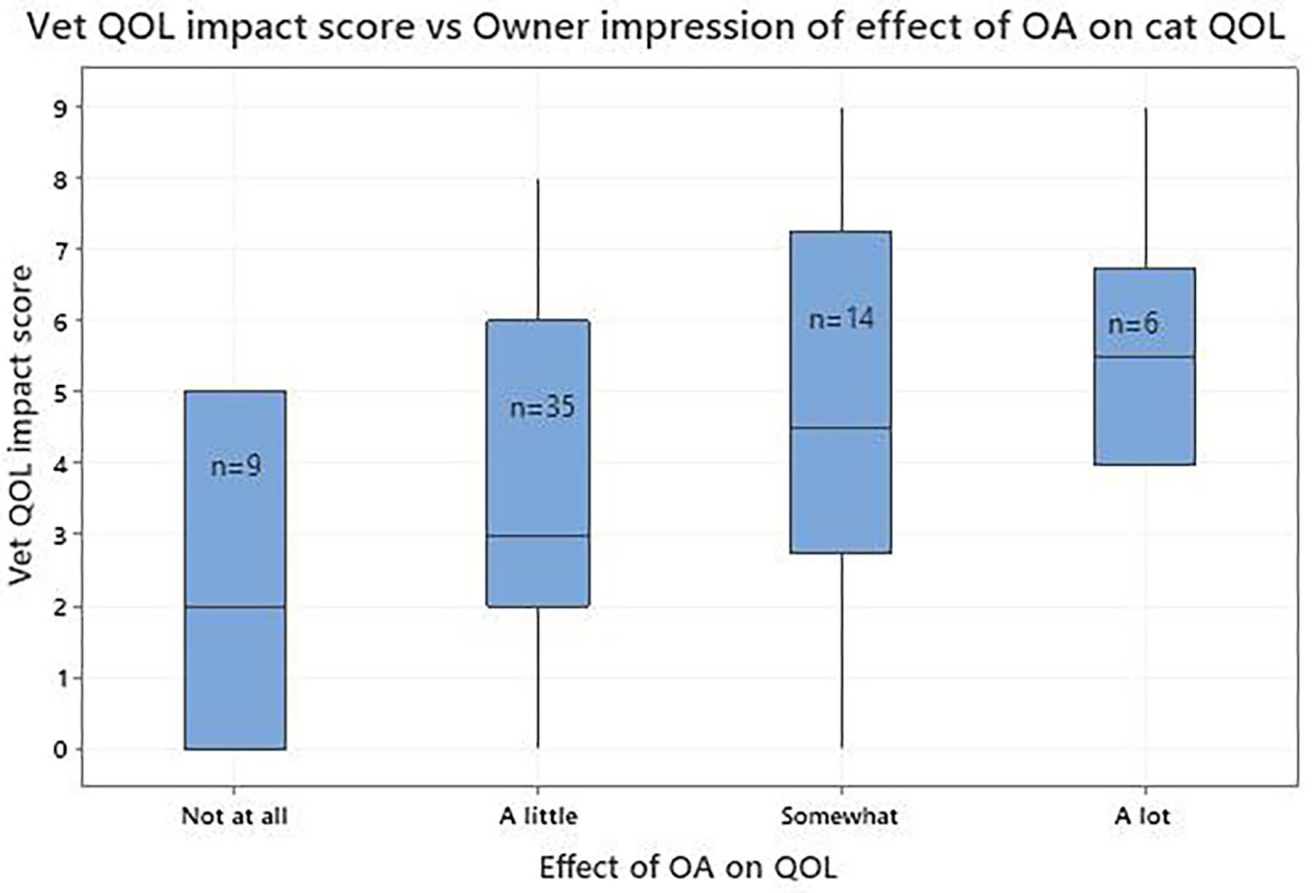
Vet-assessed quality of life (QOL) impact score (scale 0–10, where 0 is no impact and 10 is maximum possible impact) categorised by the owner's impression of QOL impact.

Seventy-one of 140 cat owners believed their cat to be in perfect health despite a veterinary diagnosis of OA. Accordingly, analysis was restricted to the 69 remaining cats, except for five whose veterinarians had not recorded QOL impact or pain scores. There was only one cat where the owner classified its QOL as very poor so, for subsequent analysis, the very poor and poor categories were combined. Figure 4; Table 6 show the agreement between the vet-assessed QOL impact score and the owner's impression of their cat's QOL. Although the number of cats in the poor/very poor category was small (4), there was a general trend with declining vet-assessed QOL impact scores as the owners' QOL impressions changed from poor through to very good. The vet-assessed QOL impact score categorised by the owner's impression of QOL impact indicated that, as the owners' impression changed from not at all to a lot, the vet QOL impact score followed this trend (Figure 5; Table 6). There were statistically significant differences between the mean vet assessed QOL impact scores for the two end categories for owner QOL status (very good and very poor/poor) and QOL impact (not at all and a lot) (Table 6).

**Table 6:** Comparison of quality of life (QOL) impact score in the vet assessment and owner impressions of their cat's QoL.

**Owner impression**		**Vet assessment**	
**Owner impression of QOL**	**Number**	**QOL impact score** ^**a**^	**Difference between end categories** ^**b**^
Poor	4	7.88 +/– 1.50	4.43 (2.19, 6.68) *P* = 0.002
Good	41	3.66 +/– 2.33	
Very good	19	3.32 +/– 2.54	
**Impact of disease on QOL**			
Not at all	9	2.22 +/- 2.22	3.44 (1.13, 5.76) *P* = 0.007
A little	35	3.43 +/- 2.38	
Somewhat	14	4.77 +/- 2.96	
A lot	16	5.67 +/- 1.86	

a *Values represent mean ± standard deviation for vet-assessed quality of life (QOL) impact score*.

b *Values represent mean (95% confidence interval) difference between vet-assessed QOL impact score for cats in end categories for owner impression of QOL (poor vs. very good) and Impact of disease on QOL (Not at all vs. A lot), along with associated P value*.

## Discussion

The aim of Objective 1 was to seek evidence for the construct validity of the VetMetrica™ instrument for use with cats suffering from OA, by testing the hypothesis that the HRQL profile of cats with different severities of OA would differ. The small group of severely affected cats restricted this to a comparison of just two groups (mild and moderate/severe) The results demonstrated that all mean HRQL domain scores for both groups fell below those of the average healthy cat (16) and there was a trend across all three domains for HRQL scores to decrease as the severity of disease increased. The difference in scores was highly significant (*p* = <0.001) in all three HRQL domains, supporting our hypothesis.

The sensitivity of the tool to disease severity represents a significant advantage over existing tools, namely the Feline Musculoskeletal Pain Index (FMPI), a disease specific clinical metrology instrument (2), and the Montreal Instrument for Cat Arthritis Testing, for use by Veterinarians (MI-CAT[V]) (3), both of which have been shown to distinguish only between normal cats and cats with OA. However, this is perhaps not surprising since both of these instruments rely on the detection of functional limitations with no regard to the emotional impact of the OA pain experience which has been described previously (30), and which causes changes in a variety of behaviours including social interaction, vocalisation and playing and hunting. In contrast, the VetMetrica™ HRQL tools are holistic measures which have a focus on the emotional as well as the physical impact of disease (12, 15) which appears to result in better discrimination of disease severity.

Furthermore, the demonstration of convergent validity between HRQL domain scores and the independently quantified vet-assessed pain and QOL impact scores supports the construct validity of the generic HRQL scale in cats with OA. An initial simple approach using Pearson correlation coefficients was adopted and the negative correlations for both vet-assessed scores and all HRQL domain scores demonstrated that as vet-assessed pain and QOL impact scores increased, HRQL domain scores decreased, providing evidence for convergent validity. All coefficients were statistically significant, but were within the range 0.20–0.39 which suggests a weak relationship between these variables (29). However, confounding factors may be that most of the cats were aged 7 years and older (Figure 1) and all but 11 had co-morbidities. Accordingly, it was decided to further investigate the relationship between vet-assessed pain and QOL impact scores and HRQL domain scores by jointly modelling the effects of age and co-morbidities to take account of these confounding factors. The regression modelling showed that for vet-assessed pain, the model was improved by including age and the number of co-morbidities. This was not unexpected since older cats are likely to be in greater pain because of the higher incidence of co-morbid conditions, many of which are likely to cause pain (15). In contrast, for vet-assessed QOL impact, the relationship with the HRQL domains was improved by including the number of co-morbidities, but not age. This concurs with previous findings which have shown that as co-morbidities increase in cats, HRQL scores decrease indicating a poorer quality of life (15). However, it is the case that all the models explained only a small percentage of the variation in the vet-assessed scores suggesting there are other factors which veterinarians use in their subjective judgements that are not part of the owner assessments. A variety of factors can affect the subjective assessment of pain by veterinarians including their age, sex and year of graduation (31–34) and these may have influenced the vet-assessed pain scores. The assessment of pain is fundamental to effective pain management so veterinarians are very conversant with pain assessment, but less so when it comes to assessment of QOL. Indeed, it could be argued that because veterinarians are not familiar with the day-to-day behaviour of their patients in the home environment they are disadvantaged when asked to assess the impact of disease on QOL. This may be an explanation for the fact that <15% of the variation in vet-assessed QOL impact scores was accounted for in the model. Nevertheless, the results support the convergent validity of the instrument, going beyond the basic correlation analysis to take account of other relevant factors (age and co-morbidities) and show that the HRQL domain scores are related to the vet-assessed scores as would be predicted.

Responsiveness is recognised to be an essential quality in any evaluative HRQL instrument but there are different views on how it should be defined and assessed (27). Broadly speaking, responsiveness has been defined as the ability to detect general change (35), which is expressed as a statistically significant change after treatment; as the ability to detect clinically important change (36), which requires a subjective judgement by a patient/carer/clinician as to what constitutes importance; and as the ability to detect true change in the construct (37), which was the definition adopted in this study. Linked to these definitions are a number of methods by which responsiveness can be assessed and the method chosen for this study was to demonstrate that the VetMetrica™ feline instrument could discriminate between those cats whose health status had improved, deteriorated or remained unchanged according to the veterinarian, using *t*-tests.

Because in some cases more than one observation per cat was used, it could be argued that the independence assumption required for the *t*-tests was not satisfied. However, the average time between observations in those cats with multiple observations was around 6 months and on that basis the authors suggest that these could be considered independent. Furthermore, the observations were divided between the three different types of change in the vet-assessed pain and QOL impact scores (decrease, no change and increase) meaning the number of observations from the same cat in any sample was limited. Also in each case, it was the change in both the HRQL domain scores and the vet-assessed pain and QOL impact scores which were used, resulting in any trend for a given cat (e.g., disease progression) being removed, and similarly any effect of systematically higher or lower scores in a given cat being accounted for.

When both vet-assessed pain and QOL scores decreased, indicating an improvement in health status, all HRQL domains showed a positive change which concurred with the assumption that the cat's health had improved. However only in Vitality and EWB domains were the changes significant. These HRQL domains tend to reflect the affective (emotional) component of the pain/QOL experience, which may be most likely to change in response to treatment, while the Comfort domain contains items relating to the impairment in physical function caused by OA. It is a limitation of this study that information regarding treatment for their OA was not reported for all cats and many, especially those with mild disease, are unlikely to have received any therapeutic intervention. Similarly, when the vet-assessed pain and QOL scores increased, indicating a deterioration in health status, there was a decrease in HRQL domain scores, but again only two domains, Comfort and EWB, reached statistical significance while the Vitality domain did not. Previously we have demonstrated more variation in the Vitality domain in healthy cats than in Comfort and EWB domains (15) which may have contributed to this finding. However, it is noteworthy that although the complete sample size was moderate (134), only 20 and 27 cats, respectively, had decreased or increased vet-assessed pain and QOL scores, with 87 cats recorded as having not changed. Thus, the lack of significance in any domain scores changes may be a consequence of small sample size.

Previously we have reported discordance between cat owners' impression of health status and that of the veterinarian, by asking the owners of cats with a veterinary diagnosis of illness, likely to affect their QOL, if their pet was “in perfect health” and in two separate field studies 29 and 26% of owners believed their cats to be in perfect health (15). In the study reported here 51% of owners of cats with a veterinary diagnosis of OA believed their cats to be in perfect health which is considerably higher. However, 63% of these cats were suffering from mild OA according to their veterinarian, and owners may not recognise the subtle signs of that degree of illness which may account for the increase. These findings highlight the need for more owner education in the field of OA recognition and management.

In this study we extended our previous more general approach by seeking to determine if, when owners accepted that their cats were not in perfect health, their impression of the QOL of their cats with OA and associated co-morbidities concurred with the veterinarian's impression of QOL impact. There was little difference in median QOL impact scores in owner reported QOL between good and very good categories which may be due to the difficulty experienced by owners when separating these. It is notable that only four owners thought their cat had a poor/very poor QOL which may be a consequence of social desirability bias (38), when owners are reluctant to admit that their cats have a less than optimum QOL because of the perceptions of others. Similarly, there was little difference in the median QOL impact scores between groups whose QOL was impacted a little and somewhat according to the owners, suggesting difficulty in distinguishing these groups. Also, a very small number of owners believed their cats to be at the ends of the scale; not affected (*n* = 8) and affected a lot (*n* = 6). Notwithstanding these observations, when the vet-assessed QOL impact scores were compared for each of the owner reports for QOL and QOL impact at the extremes of the scales there was a statistically significant difference, validating each of these global assessments and demonstrating concurrence between veterinarian and owner impression of the impact of OA on HRQL.

The validity of any HRQL instrument must be supported by a body of evidence which demonstrates that it is valid for particular purposes, with defined populations and in specified contexts (22) and the work reported in Objective 1 has—using known groups and convergent validity approaches—provided evidence for the construct validity of the feline VetMetrica™ generic instrument for the measurement of HRQL of cats with OA–. Furthermore, if responsiveness can be considered to provide evidence of longitudinal validity (27) then the results of Objective 2 also add to the body of evidence supporting the instrument's validity for use with this new population.

The study has provided initial evidence for the responsiveness of the instrument to clinical change in cats with OA, an essential quality in an instrument designed for clinical evaluation purposes. An important next step will be to facilitate interpretation of score changes in a clinical or research context by calculating a minimum important difference (MID) for cats with OA. The MID has been defined as “*the smallest difference in score in the outcome of interest that informed patients or informed proxies perceive as important, either beneficial or harmful, and which would lead the patient or clinician to consider a change in the management”* (39). Previously we have reported MID values for Vitality, Comfort and EWB domains in cats suffering from a variety of chronic conditions (16), but the MID is not a fixed property of the scale and will vary according to the population and the context in which it is being used. A population of cats with OA is likely to be less heterogeneous than that used previously and so the MID values are likely to differ. Given the number of clinical trials being conducted currently to develop new therapeutic interventions for feline OA, the determination of MID will assume increasing importance for the calculation of sample size as this depends on the magnitude of the difference investigators consider clinically important (40).

In conclusion, Objectives 1 and 2 have provided additional evidence to support the validity of the feline VetMetrica™ generic HRQL instrument and demonstrated its value as an outcome measure in trials designed to demonstrate the efficacy of therapeutic interventions for OA in the cat. Objective 3 has shown that while a high proportion of owners of cats with OA are reluctant to admit their cats are in ill health, those that do are in agreement with their veterinarian's impression of the impact of the disease on their cats' QOL.

## Data Availability Statement

The original contributions presented in the study are included in the article/Supplementary Material, further inquiries can be directed to the corresponding author/s.

## Ethics Statement

The animal study was reviewed and approved by University of Glasgow Ethics Committee. Written informed consent was obtained from the owners for the participation of their animals in this study.

## Author Contributions

VD, CN, AN, JR, ES, and MLW-O: Conceptualisation. ND, AG, CN, and JR: Provision of data. VD and ES: Statistical analysis. All authors contributed to the article and approved the final version, writing, reviewing, and editing.

## Funding

This study received funding from NewMetrica Ltd.

## Conflict of Interest

JR is a Director of NewMetrica Research Ltd, a company that develops the VetMetrica™ instruments. CN and JR were employed by NewMetrica Research Ltd. At the time the study was performed, ND was undertaking a post-graduate studentship, also funded by Royal Canin, a division of Mars Petcare. Since October 2020, ND has been employed by International Cat Care, but also holds a part-time post-doctoral research position at the University of Liverpool, funded by Royal Canin. AG is an employee of the University of Liverpool whose position is funded by Royal Canin. He has received financial remuneration and gifts for providing educational material, speaking at conferences, and consultancy work. However, neither Royal Canin nor any other part of the Mars Petcare business were involved in either study design and execution or in data analysis and interpretation. This study received funding from NewMetrica Ltd. The funder had the following involvement with the study: Both JR and CN were involved in study design, data collection, decision to publish and preparation of the manuscript. The remaining authors declare that the research was conducted in the absence of any commercial or financial relationships that could be construed as a potential conflict of interest.

## Publisher's Note

All claims expressed in this article are solely those of the authors and do not necessarily represent those of their affiliated organizations, or those of the publisher, the editors and the reviewers. Any product that may be evaluated in this article, or claim that may be made by its manufacturer, is not guaranteed or endorsed by the publisher.
